# In-vitro analysis of selective nutraceuticals binding to human transcription factors through computer aided molecular docking predictions

**DOI:** 10.6026/97320630012354

**Published:** 2016-10-17

**Authors:** Mohammad Teimouri, Muhammad Junaid, Shoaib Saleem, Abbas Khan, Arif Ali

**Affiliations:** 1 Department of Biochemistry, Huazhong University of science and Technology, China; 2 Department of Bioinformatics and Biostatistics, Shanghai Jiao tong University, Shanghai, China; 3 3Center for Biotechnology & Microbiology, University of Swat, Swat Khyber, Pakhtunkhwa, Pakistan

**Keywords:** In-vitro analysis, Nutraceuticals, Human transcription factors, computer aided molecular docking

## Abstract

The contest of cancer couldn’t be completed without novel drug with novel modes of action, improved efficacy and acceptable pharmacokinetic properties. Transcription factors are attractive targets to develop anti-cancerous drugs. 6-Gingerol, Anethol analogues, Capsaicinoids, Curcumin, Dibenzoylmethane, Diosgenin, Eugenol, Gambogic acid, Thymoquinone, Ursolic acid, Xanthohumol, Zerumbone are the promising nutraceuticals that help in the prevention of cancer. These nutraceuticals showed promising activity in invitro tests. In this study In-silico tools were applied to confirm the activity of these nutraceuticals against the transcription factors including Nuclear Factor-Kappa B (NF-κB), AP-1, NRF2, PPAR-γ, β-catenin/Wnt and Sonic Hedgehog. This studied followed molecular docking based approach to verify the in-vitro activities of the said nutraceuticals against the cancer. Molecular Docking based approached provide a path towards the identification of novel ligands against these transcription factors. Based on the interaction of Cardamoninand capsaicin it was found to have an influencing role against the transcription factor like NF-κB andPPAR-γ. The interaction of Cardamoninwith NF- κBand capsaicinwith PPAR-γ provide a way toward structure-based virtual screening to identify novel ligands against the targets which could be very help full in successful chemotherapy of cancer. This study delivers structural features of nutraceuticals and its interactions against different transcription factors and gives a theoretical entry to use these compounds as a potential inhibitor against the transcription factors involved in cancer.

## Background

Cancer is a complicated disease which has caused large number of deaths throughout the world. It is considered as the combine effect Environment and genome. Mutated genes or somatic mutation is counted for only 5-10% of the cancer. The remaining 90-95% of the cancer incidences is due to the life style and environmental factors of an individual. Almost 30% of all cancers have been attributed to tobacco smoke, 35% to diet, 14–20% to obesity, 18% to infections while environmental pollutions and radiation contribute 7% only. These mechanisms are evident by many researches. One process that seems to be common to all these risk factors is inflammation [1,2]. However, large number of transcription factors is also associated with different types of cancers and their probable role has been identified in the development, progression of cancer and tumorigenesis. Tumor development and cell proliferation activity of PPARγ has long been investigated and reported mostly in colon cancer cell lines, colonic tumors and normal colonic mucosa [3]. Nf- κB has been reported along with PPARγ in the colon cancer and pancreatic cancer [4]. Activator protein-1 (AP-1) transcription factor has been reported to be associated with breast cancer [5]. In cancer cell Nrf2 has also been testified to augment cell proliferation through the inhibition of apoptosis, while beta-catenin has been verified for their malignancies role in different types of cancers such as medulloblastoma pilomatricomas, hepatocellular carcinoma, colon cancer, ovarian cancer, melanoma, prostate cancer and endometrial cancer [6,7]. Many attempts were made for the successful chemotherapy of cancer but still failed. The failure in the chemotherapy of cancer is due to our narrow focus which could be a single gene, single gene product, or a single metabolic pathway. These narrow targets for such a complex disease would not favor the successful chemotherapy. An alternative to pharmaceuticals researchers are now focusing on nutraceuticals which has overcome the problem of specificity. Spices are known for their flavor, taste, and color in the food and have been used for thousands of years, they are not usually recognized for their medicinal value [8]. A number of nutraceuticals, have shown potential to reduce cancer incidences by inducing apoptosis by targeting multiple pathways [9]. The term nutraceuticals was defined by Stephen DeFelice in 1989 as “a food (or part of a food) that provides medical or health benefits, including the prevention and/or treatment of a disease” [10,11]. Despite the fact that nutraceuticals is using in food, it also shows great potential for modulating multiple targets such as transcription factors including peroxisome proliferator-activated receptor (PPARγ ), STAT3, activator protein (AP-1), NRF-2, HIF-1α and NF-κB involved in tumor progression [12]. Potential Nutraceutical compounds such as curcumin, resveratrol, selenium, and vitamin D are found to have inhibitory effect against the transcription factors. Curcumin were primarily found against Wnt/β-catenin and NF-κB [13]. For this study we selected some of the nutraceuticals and transcription factors based on their in-vitro study reports aims to confirm these in-vitro tested nutraceuticals against the transcription factors involved in cancer. Here we applied computational tool, molecular docking, to confirm the in-vitro tests of these nutraceutical against the cancer. Whether or not these nutraceutical showing any activity against the transcription factors.

## Methodology

Around 15 different nutraceutical active compounds were docked against the transcription factors primarily involved in cancer development. Ligands and receptor preparation along with docking was carried out using MOE (Molecular Operating Environment), while Discover Studio visualizer 4.5 Client (http://accelrys.com/) and Pymol visualization software (https://www.pymol.org) was used to visualize the results.

### Ligand’s searching and database preparation

The 3D structures of different nutraceuticals were retrieved from chemspider (http://www.chemspider.com/), drugbank (http://www.drugbank.ca/) and some were drawn through Chemdraw 12. Different nutraceuticals were docked in this study. The database of these known nutraceuticals was prepared using MOE. An .mdb file was generated and all the ligands were added to that. Before the docking the protonation and energy minimization of the 3D structures were carried out using MOE (Molecular Operating Environment) [14] .

### Retrieving and Refinement of Receptor Proteins

**Figure 2:**
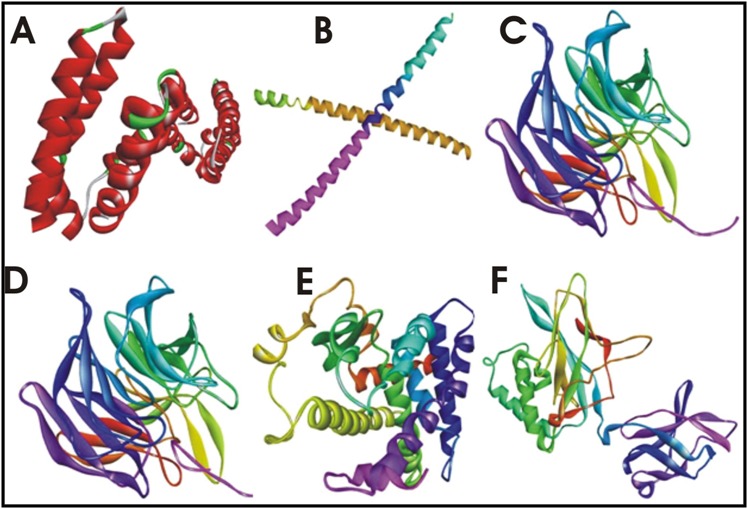
A: showing the structure of Nuclear Factor-Kappa B (NF-κB) (1SVC), B: showing the structure of AP-1(1FOS), C: showing the structure of NRF2 (2FLU), D: showing the structure of PPAR-γ (1ZGY), E: showing the structure of β-catenin/Wnt (3FQN), F: showing the structure of Sonic Hedgehog (3MXW).

The 3D structures of different important targeted proteins were retrieved from RCSB [15]. For good output low resolution X-ray structures were retrieved from the database. Before the docking the protonation and energy minimization of the 3D structures were carried out using MOE (Molecular Operating Environment). A list of receptors used in this study along with their 3D structure is given in the Figure 2.

### Molecular Docking

The docking of nutraceuticals against the given proteins was carried out on using MOE [14]. The docking was carried out against each protein separately. Active site Finder tool of MOE was used to identify and calculate active sites in the receptor molecule from the 3D atomic coordinates of the receptor. By default, all calculated sites were appeared as selected. Before the docking a database of these ligands was prepared using MOE. The parameters were set (Re-scoring function: London dG , placement: triangle matcher, Retain: 10, Refinement: Force field, and Re-scoring 2: London dG). Docking program of MOE provides correct conformation of the ligand so as to obtain minimum energy structure. After docking, S score was considered the criteria to select best conformation for nutraceuticals and these were then further studied to analyze the hydrogen bonding/π-π interactions through LigX tool of MOE.

## Results

**Figure 3:**
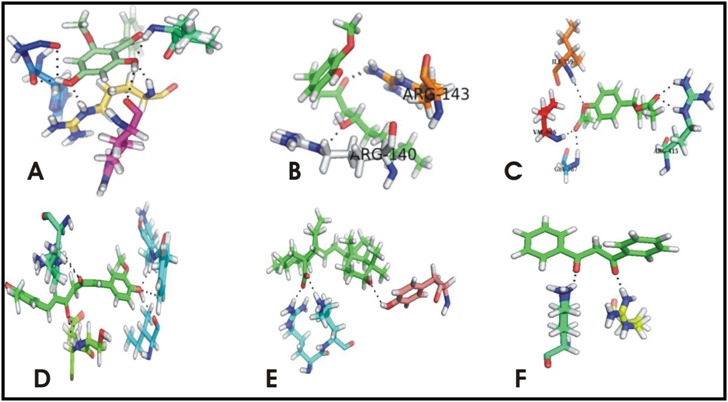
A: Showing Cardamonin in interaction with NFK-B, B: Showing 6-[Gingerol] in interaction with AP-1, C: Showing 1'- Acetoxychavicol acetate in interaction with NRF2, D: Showing Capsaicin in interaction with PPAR-γ, E: Showing Ursolic acid in interaction with β-catenin/Wnt, F: Showing Dibenzoylmethane in interaction with Sonic Hedgehog.

Around 30 different conformations were allowed to each ligand. These conformations were stored then and their score was used to select the best conformations. Ligands with good scoring were selected for further hydrogen bond, covalent bond and other interactions analysis. The 2D depiction of the best compounds was saved and reported. MOE provide a best docking tool which is using S-score to identify good affinity compounds. After the docking of these nutraceuticals against each receptor, the results of each ligand were checked against the given receptors. The interaction of all the ligands against each protein was found to have good activity but only with the best of all was selected for 2D and 3D visualization. Cardamonin was found to have the best of all the docked ligand against the NFK-B transcription factor. It was found to have -13.8506 docking score the best score of all, with 6 hydrogen bonds against NFK-B. 1'-Acetoxychavicol showed good activity against NRF2. The best docking score of 1'-Acetoxychavicol was -9.53229 against NRF2 with the formation of 5 hydrogen bonds. PPAR-γ was paralyzed by the activity of Capsaicin. The docking score Capsaicin -12.5873 in association with formation of 6 hydrogen bonds was observed. However the activity of Ursolic acid and Dibenzoylmethane was found effective against β- catenin/Wnt and Sonic Hedgehog respectively. The best docking score against these proteins were -9.6406. 6-[Gingerol] was found in good interaction with AP-1 with the docking score -10.2340 coupled with only 2 hydrogen bonds. The results are showing that Capsaicin, Cardamonin, 1'-Acetoxychavicol, Ursolic acid, 6- [Gingerol] and Dibenzoylmethane showed influential role against the different transcription factors involved in cancer. The significant activity of these nutraceuticals against the transcription factors is also providing a choice of combinatorial chemotherapy. The best docking score, RMSD, Number of hydrogen bonds formed by these ligands against the given receptors are shown in Table 1. The binding affinity of all these against the receptor of all the ligands are shown in the Figure 3.

## Conclusion

Cancer remains one of the major fatal diseases, which causes number of deaths annually around the world. Earlier in-vitro tests exposed that nutraceuticals could be the potential use against the transcription factors primarily involved in cancer. Here the molecular docking approach also revealed that the interaction of these different nutraceuticals is showing significant activity against the cancer. The information provided by the binding modes of these nutraceuticals facilitates the synthesis and testing of nutraceuticals against the transcription factors. Nutraceutical like Capsaicin, Cardamonin, 1'-Acetoxychavicol, Ursolic acid, 6- [Gingerol] and Dibenzoylmethane are showing the promising role against the cancer therapy. Here we also concluded that these nutraceutical not only showed excellent activity against the specific transcription factors but all showed a promising role against each transcription factors. This in silico study delivers structural features of nutraceuticals and its interactions against different transcription factors and gives a theoretical entry to use this compound as a potential inhibitor against the transcription factors involved in cancer. Concluding remarks would suggest that nutraceuticals are of great interest in case of successful cancer chemotherapy

## Figures and Tables

**Table 1:** The best docking score of each receptor with their hydrogen bonds and interacting residues

S. No	Receptor	S-Score	RMSD	Hydrogen Bonds	Interacting Residues
1	NF-κB	-13.8506	1.2362	6	Arg57, His67, Glu63, Gly68, Ser66, Arg59
2	AP-1	-10.234	1.5695	2	Arg140, Arg143
3	NRF2	-9.53229	1.3637	5	Gly509, Arg415, Gly462, Gly367, Val606
4	PPAR-γ	-12.5873	2.9742	6	Glu343, Arg288, Isoleu262, Glu295, Glu291, His266
5	β-catenin/Wnt	-9.6406	4.0509	5	Arg65, Lys66, Tyr159, Leu156,

**Figure 1:**
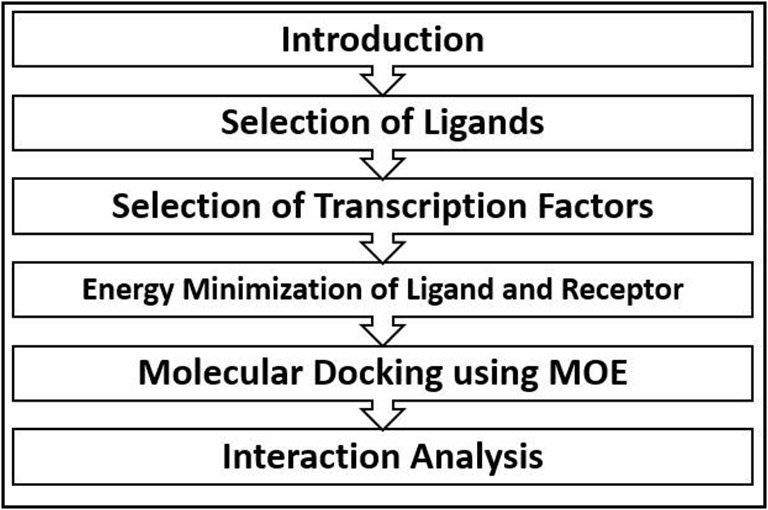
Graphical abstract of the work illustrated using a flowchart
